# Lesion based diagnostic performance of dual phase ^99m^Tc-MIBI SPECT/CT imaging and ultrasonography in patients with secondary hyperparathyroidism

**DOI:** 10.1186/s12880-017-0235-3

**Published:** 2017-12-12

**Authors:** Panli Li, Qiufang Liu, Daoqiang Tang, Yinyan Zhu, Lian Xu, Xiaoguang Sun, Shaoli Song

**Affiliations:** 1 Department of Nuclear Medicine, Ren Ji Hospital, School of Medicine, Shanghai Jiao Tong University, 160 Pujian Road, Pudong District, Shanghai, 200127 China; 2 SJTU-USYD Joint Research Alliance for Translational Medicine, Shanghai, 200240 China; 3 Department of Pathology, Ren Ji Hospital, School of Medicine, Shanghai Jiao Tong University, Shanghai, 200127 China

**Keywords:** ^99m^Tc-MIBI SPECT/CT, Secondary hyperparathyroidism, Serum PTH level, Lesion diameter

## Abstract

**Background:**

We aimed to evaluate the diagnostic performance of ^99m^Tc-MIBI SPECT/CT and ultrasonography in patients with secondary hyperparathyroidism (SHPT), and explored the factors that affect the diagnostic performance.

**Methods:**

^99m^Tc-MIBI SPECT/CT and ultrasonography were performed in 50 patients with SHPT within 1 month before they underwent surgery. Imaging results were confirmed by the pathology. Pearson correlation analysis was used to determine the correlation of PTH level with clinical data. The optimal cutoff value for predicting positive ^99m^Tc-MIBI results was evaluated by ROC analysis in lesions diameter.

**Results:**

Forty-nine patients had a positive ^99m^Tc-MIBI imaging results and 39 patients had positive ultrasonography results. The sensitivities of ^99m^Tc-MIBI and ultrasonography were 98.00% and 78.00%, respectively. A total of 199 lesions were resected in 50 patients. Among them, 183 lesions were proved to be parathyroid hyperplasia. On per-lesion basis analysis, the sensitivity and specificity of ^99m^Tc-MIBI and ultrasonography were 59.34% and 75.00% vs 46.24% and 80.00%, respectively. The Pearson correlation analysis showed that the serum AKP and PTH level had a significant linear association (*r* = 0.699, *P* < 0.001). The lesion diameter was a statistically significant predictive factor in predicting positive ^99m^Tc-MIBI SPECT/CT. The optimal cutoff value for predicting positive ^99m^Tc-MIBI results evaluated by ROC analysis in lesions diameter was 8.05 mm.

**Conclusion:**

Dual phase ^99m^Tc-MIBI SPECT/CT imaging had a higher sensitivity in patients with SHPT than ultrasonography. Therefore, using ^99m^Tc-MIBI positioning the lesion could be an effective method pre-surgical in patients with SHPT.

## Background

Secondary hyperparathyroidism (SHPT) occurs due to a progressive increase in the level of parathyroid hormone (PTH) in diseases that affect the metabolism of calcium or phosphorus and is a common complication in patients with chronic kidney disease [1, 2]. Patients with severe SHPT often develop high turnover bone disease, which can cause bone pain and skeletal fractures risk [3]. SHPT is also associated with an increased risk of cardiovascular calcification and mortality [4]. Traditional therapies such as administration of calcium salts and vitamin D are limited by hypercalcemia, hyperphosphatemia and lack of long-term efficacy [5].

Surgical parathyroidectomy remains the only option in 5% to 10% of patients with chronic renal failure who are treated with long-term dialysis and fail to respond to medical therapy [6, 7]. Successful surgical treatment can reduce the PTH levels and improve the clinical symptoms such bone pain. Different from primary hyperparathyroidism (PHPT) in which patients generally have only one lesion [8], patients with SHPT have multiple lesions [9]. The unguided bilateral neck exploration is effective in 90–95% patients [10]: surgical failure is due to ectopic glands and undetected multi-glandular disease. Surgical outcome is often unfavorable, resulting in persistent or recurrent hyperparathyroidism in 10–30% cases [11]; therefore, pre-surgical localization is necessary in patients with SHPT.

Parathyroid glands can be detected with multiple modalities, such as imaging, high-resolution (7.0–14.0 MHz) ultrasonography (USG), thin-section CT and MRI [12]. USG is an advantageous modality as a non- radiation emitting, and widely available technique in clinic. ^99m^Tc-methoxyisobutyl isonitrile (^99m^Tc-MIBI) is a common radiopharmaceutical used in parathyroid imaging. Dual-phase parathyroid imaging with ^99m^Tc-MIBI currently represents the current diagnostic modality for parathyroid glands. The diagnostic performance of ^99m^Tc-MIBI SPECT/CT in patients with SHPT showed a higher sensitivity and specificity compared with ultrasonography in detecting hyperplastic glands on a per-patient-based analysis [9, 13].

There were already some studies have reported the diagnostic performance of ultrasonography and ^99m^Tc-MIBI dual phases imaging. But the results of sensitives had a vary difference. The sensitives of ultrasonography and ^99m^Tc-MIBI dual phases imaging had a wide range of 35.9–91.5% and 36.6–66%, respectively [14–16]. Now we study the diagnostic performance of ^99m^Tc-MIBI SPET/CT and ultrasonography in patients and lesions with SHPT were all studied, to explore the factors that affect the diagnostic performance of SPECT/CT, such as PTH level and diameter. The patients with secondary hyperparathyroidism that all had renal failure. Patients were undergoing both ^99m^Tc-MIBI dual phases SPECT/CT imaging and ultrasonography before surgery.

## Methods

### Patients characteristic

We retrospectively studied the patients who underwent parathyroid ^99m^Tc-MIBI imaging for elevated PTH level and ultrasonography for existing abnormal parathyroid between December 2012 and January 2017 in Ren Ji Hospital, School of Medicine, Shanghai Jiao Tong University. Medical record review included patient demographics (gender, age, dialysis vintage), clinical history, imaging, laboratory values (calcium, phosphorus, parathyroid hormone, alkaline phosphatase), and operative and pathological results and outcomes. Fifty pathologically confirmed secondary hyperparathyroidism patients were included in this study.

### Imaging examinations

All patients with SHPT received an intravenous injection of 740 MBq of ^99m^Tc-MIBI. Early parathyroid imaging was obtained 20 min post injection, and a delayed parathyroid imaging was obtained 2 h post the injection. SPECT/CT integrated imaging was performed at 2 h immediately after the delayed planar image. The imaging acquisition was using Philips precedence 16 (Philips, Medical Systems, Netherlands). A 256 × 256 matrix was used and 32 20-s projections were acquired over 360°. Imaging data were reconstructed using a three-dimensional iterative algorithm. Images were smoothed with a three-dimensional spatial Gaussian filter. CT acquisition parameters were as follows: tube current, 250 mA; and tube voltage, 120 kV. For CT data reconstruction, a 3-mm slice thickness with 2-mm slice increment was used. Both SPECT and CT 3-mm slices were generated using an Astonish bone application package (Philips) and were transferred to a picture archiving and communication system after generation of DICOM files. SPECT/CT images were fused using the Syntegra software (Philips). Ultrasonography (USG) was performed using the Technos DU8 scanner (Esaote SpA, Genoa Italy) with an 8 - 13MHZ LA523 linear-array transducer.

### Image analysis

**Fig. 1:**
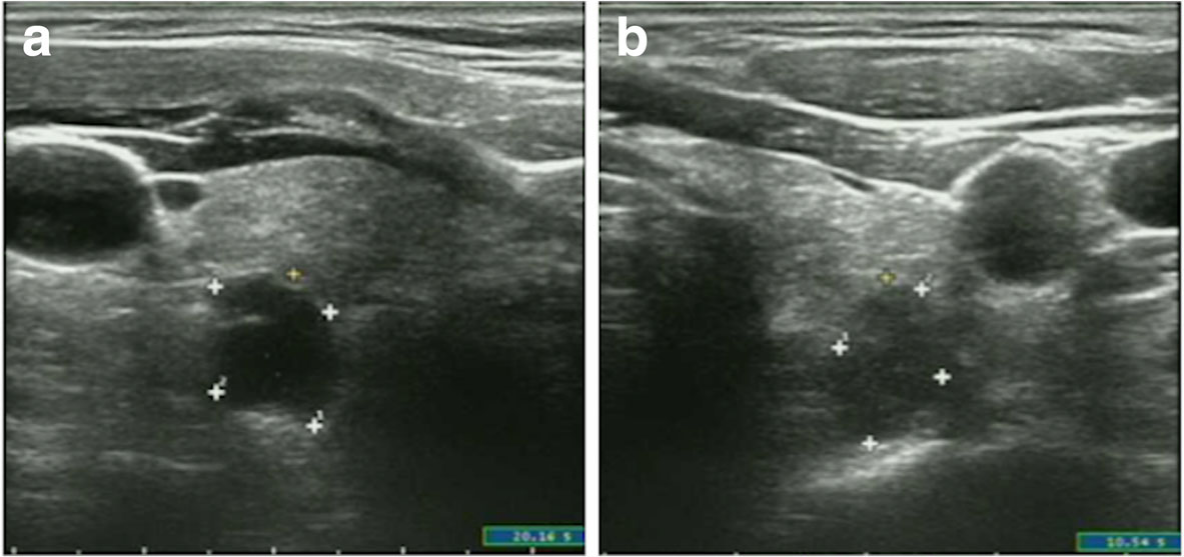
An elderly patient with secondary hyperparathyroidism performed the ultrasonography. The image **a** showed an enlarged parathyroid gland on the left, with a size of 13.5 mm × 10.8 mm. The image **b** showed the right enlarged parathyroid gland, with a size of 8.2 mm × 12.8 mm

The imaging results were evaluated by visual analysis and the uptake value of lesions were judged by semi-quantitative visual analysis. SPECT/CT images were analyzed by two experienced nuclear medicine physicians who were blinded to the laboratory, surgical, and pathological results. Abnormal ^99m^Tc-MIBI uptake was considered to be positive on visual analysis, and the different uptake value of each lesion was graded to three levels through the calculation of the tumor to background ratio (TBR) for the delayed phase. These areas were scored for activity on a three-point scale: 1 = slight uptake (1 < TRB ≤ 2), 2 = medium uptake (2 < TRB ≤ 3), 3 = high uptake (TRB > 3) [17] (Fig. 1). A positive parathyroid imaging result was increased uptake of ^99m^Tc-MIBI on the delayed image compared to the early-stage image, with precise localization of the focus on the delayed planar image by SPECT/CT; negative parathyroid scan had no ^99m^Tc-MIBI increased uptake on the delayed image compared to background.

The diameters of excised parathyroid adenoma, hyperplasia, or parathyroid carcinoma were measured by SPECT/CT. In positive ^99m^Tc-MIBI cases, SPECT/CT located not only the lesion with ^99m^Tc-MIBI on planar imaging but also measured the diameters of tumor.

### Statistical analysis

Data are shown as mean ± standard deviation (SD). Semi-quantitative analysis of ^99m^Tc-MIBI SPECT/CT was compared with the uptake value sum of a patient and the PTH level. Pearson’s correlation co-efficient was used for statistical analysis. Comparisons of continuous variables between two groups were performed using the *t* test. The Chi-square test was used for categorical variables. The optimal cutoff value of lesion diameter for predicting positive ^99m^Tc-MIBI imaging was evaluated using receiver-operating characteristic (ROC) analysis. *P* value less than 0.05 was considered significant in this study. Statistical analysis was performed using the MedCalc version 15.6 statistical software (MedCalc Software bvba, Ostend, Belgium) and the IBM SPSS version 22.0 statistical software (IBM, Armonk, USA).

## Results

**Fig. 2:**
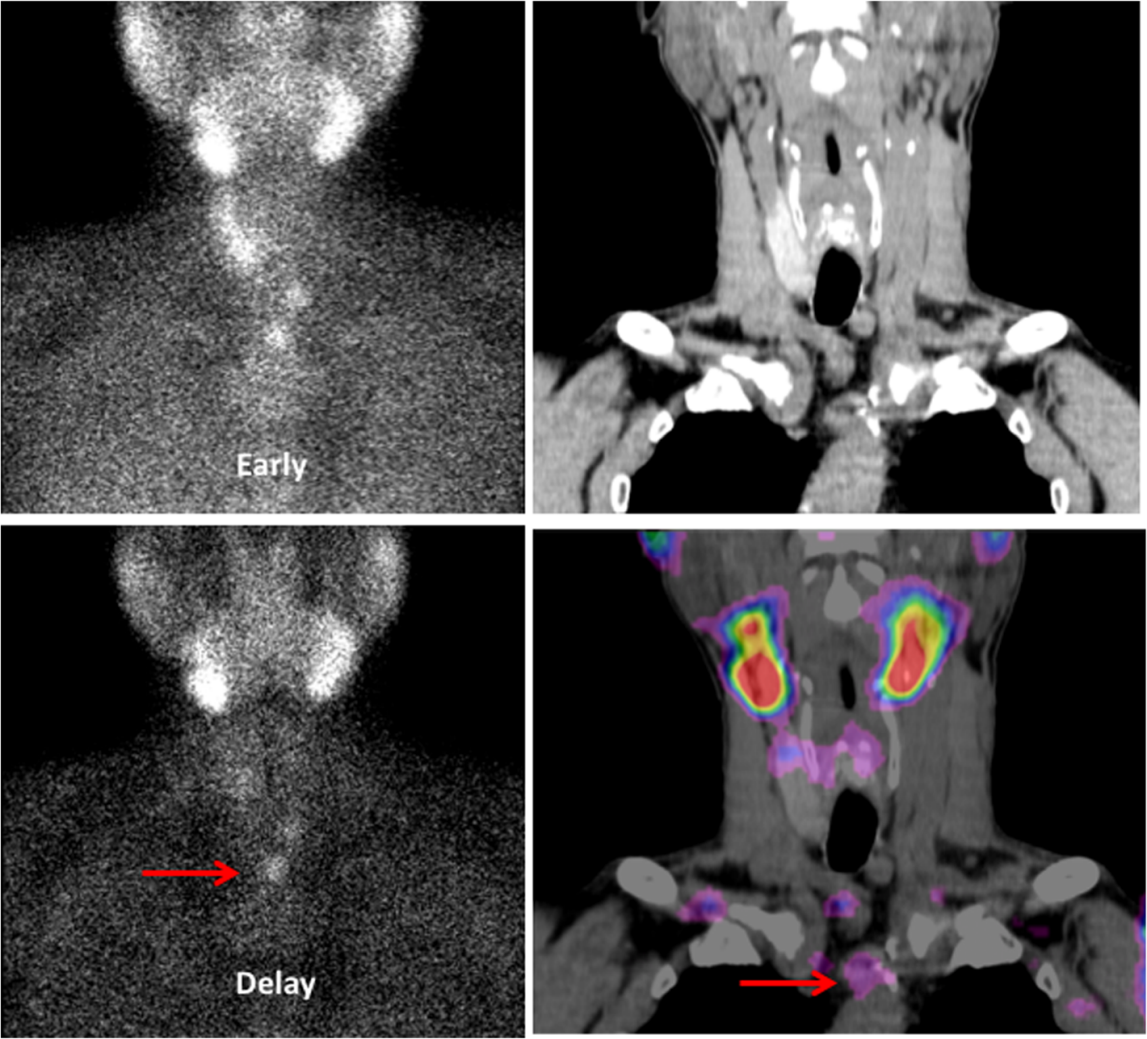
A middle-aged patient was affected by SHPT. A dual-phase ^99m^Tc-MIBI planar imaging was performed before surgery. Early and delayed phase imaging showed an ectopic parathyroid within the upper mediastinum (an arrow). ^99m^Tc-MIBI SPECT integrated CT found the lesion located in front the aortic arch. The histology result confirmed the parathyroid hyperplasia

The clinic pathological characters and ^99m^Tc-MIBI data of the 50 patients with SHPT who underwent surgery are summarized in Table 1. For the patients included in the study, the primary diseases of all patients were chronic renal failure. And among them, there were 42 patients with peritoneal dialysis or hemodialysis. Among the 50 patients, 49 (98.00%) patients had a positive ^99m^Tc-MIBI imaging, and only 1 patient had a negative ^99m^Tc-MIBI imaging. ^99m^Tc-MIBI imaging found an ectopic parathyroid in one patient (Fig. 2). For ultrasonography, there were 39 (78.00%) patients were found had at least one increased parathyroid. In patients with known serum calcium and phosphorus levels, 34.00% (17/50) and 76.00% (38/50) patients showed higher serum calcium and phosphorus levels, respectively.

**Table 1:** The clinic pathological characteristic and serological indicators data of all the 50 patients with SHPT included

Included Patients		
Sex	Female	26
	Male	24
Ages (year)	Mean ± SD	50.82 ± 12.62
Peritoneal dialysis	Number	10
	Time(years)	7.44 ± 2.99
Hemodialysis	Number	32
	Time(years)	8.78 ± 4.35
^99m^Tc-MIBI result	Positive	49
	Negative	1
US result	Positive	39
	Negative	11
Bone Pain	Yes	36
	No	14
PTH level (ng/ml)	Mean ± SD	1806 ± 867.04
AKP level (U/L)		477.90 ± 381.40
Scr level (μmol/L)		915.70 ± 289.60
Ca level (mmol/l)		2.49 ± 0.38
P level (mmol/l)		2.08 ± 0.60

*Abbreviations*: *NA* not announcementm, *PTH* parathyroid hormone, *AKP* alkaline phosphatase, *Scr* serum creatinine, *Ca* calcium, *P* phosphorus

**Fig. 3:**
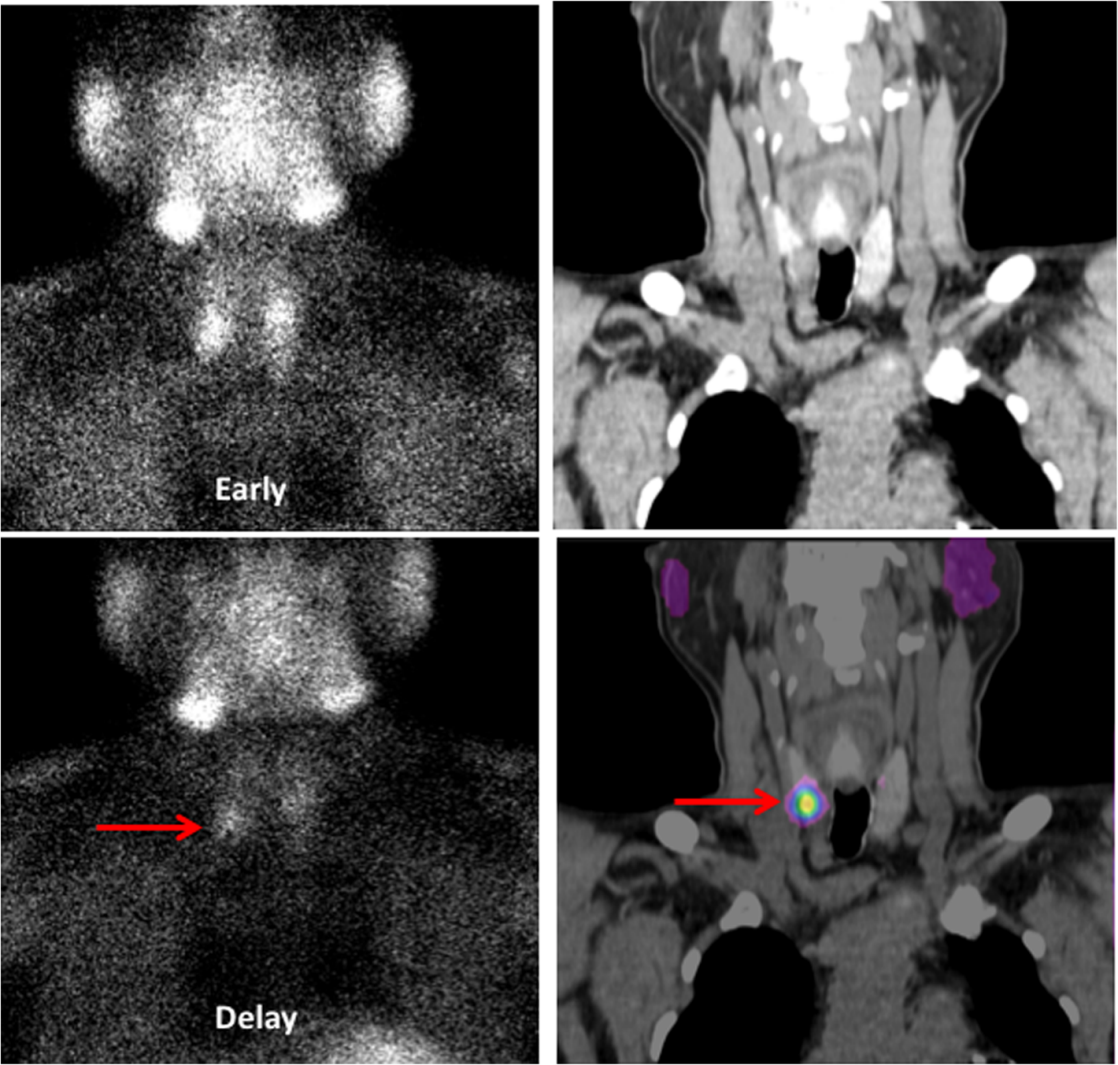
A old age patient was affected by SHPT. A dual-phase ^99m^Tc-MIBI imaging was performed. Early and delayed phase imaging showed two enlarged parathyroid glands located superior the right thyroid glands (an arrow). ^99m^Tc-MIBI SPECT/CT imaging depicting the biggest focus located superior the right thyroid gland. The histology confirmed the lesion was a parathyroid adenoma

**Fig. 4:**
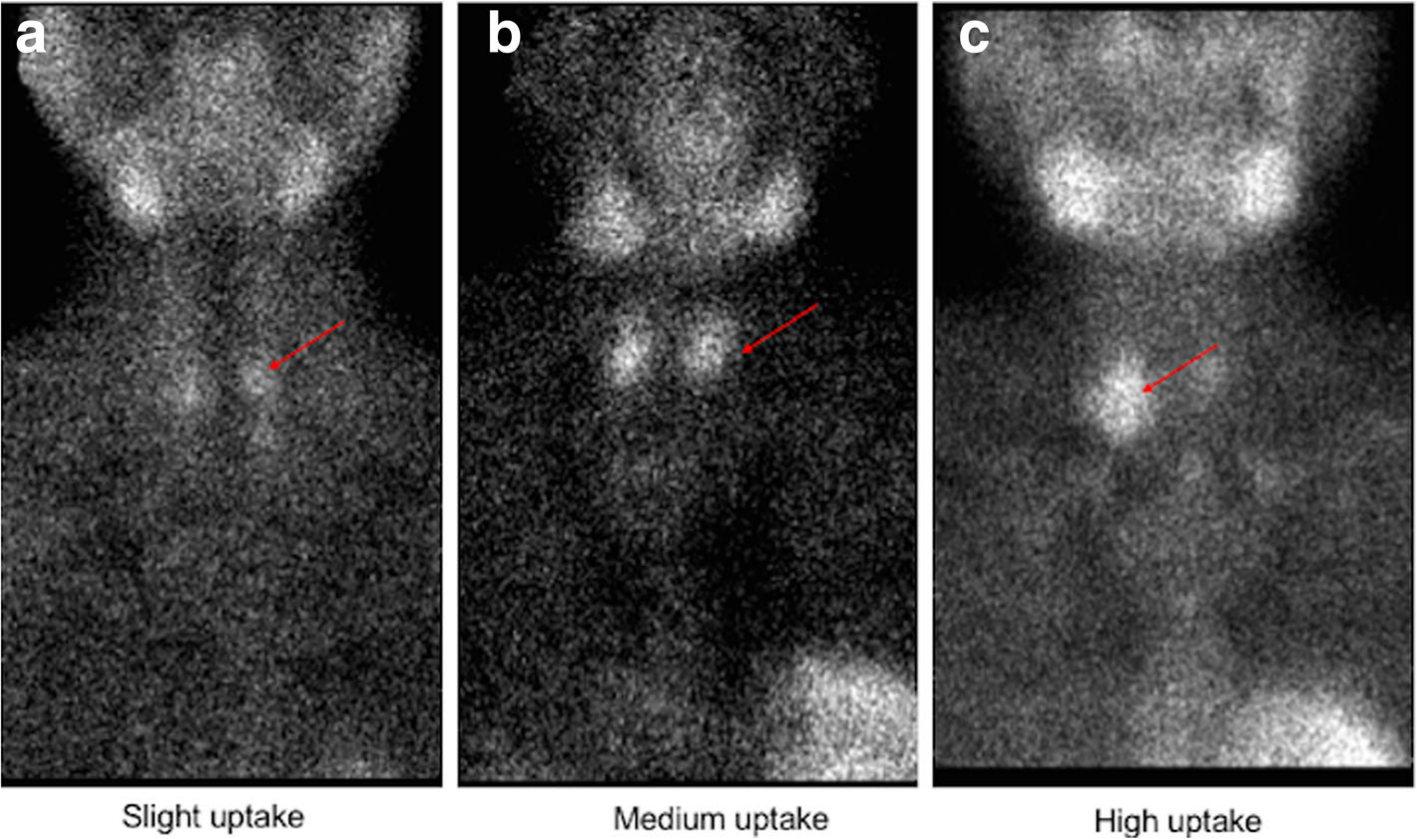
The three-point scale the score of lesions were judged by semi-quantitative visual analysis . The image **a** represent slight uptake (TBR = 1.61) scores one point, the image **b** show medium uptake (TBR = 2.26) scores two points, the image **c** show high uptake (TBR = 3.58) scores three points. The red arrow refers to the parathyroid glands with the MIBI uptake

In 50 patients who underwent surgery, two patients had parathyroid subtotal resection, 48 patients had complete parathyroid resection and autologous transplantation. A total of 199 glands were resected in the surgery of the 50 patients. One hundred and eighty-three glands were proved to be parathyroid lesions. Four glands were confirmed to be normal parathyroid glands, twelve were lymph nodes. In 100 and 83 confirmed lesions, 179 were parathyroid hyperplasia, and 4 lesions in 3 patients were confirmed to be parathyroid adenoma (Fig. 3). Among the fifty patients, the pathological results revealed one lesion from one patient (2%), two lesions from 4 patients (4%), three lesions from 6 patients (12%), and four lesions from the rest 39 patients (78%). ^99m^Tc-MIBI imaging showed 112 positive lesions in 50 patients, and 108 lesions confirmed to be true positive. For the 4 false positive lesions found in three patients, two lesions were confirmed to be thyroid tissue, two lesions were lymph nodes. Seventy-four lesions found in 39 patients showed negative ^99m^Tc-MIBI uptake but were the pathological confirmed to be parathyroid hyperplasia or adenoma. On per-lesion basis analysis, the sensitivity and specificity of ^99m^Tc-MIBI were 59.34% and 75.00%, respectively. Eighty-nine enlarged lesions in ultrasonography, 86 lesions were confirmed to be true positive. Ninety-seven normal size lesions were pathological confirmed to be parathyroid hyperplasia. Therefore, on per-lesion basis analysis, the sensitivity and specificity of ultrasonography were 46.24% and 80.00%, respectively. A representative ultrasonography imaging showed in the Fig. 4.

Abnormal ^99m^Tc-MIBI uptake was considered to be positive on visual analysis, and the different uptake strength of each lesion was graded to three levels. Each lesion was scored according to ^99m^Tc-MIBI uptake level. And then the lesions number of each patient and lesion uptake value scores of one patient were grouped for the 50 patients included; the numbers of patients in each group were listed in Table 2. Furthermore, for these patients, the number of lesions for each patient was counted on the basis of the pathological results. The correlation between PTH level and laboratory test items such as calcium, phosphorus and alkaline phosphatase were analyzed. Pearson correlation results showed a significant linear association between the serum AKP level and PTH level (*r* = 0.699, *P* < 0.001). The lesions diameter and the number of lesions were confirmed by pathological results, and the lesion uptake value scores of each patient showed no significant linear association with PTH levels.

**Table 2:** The lesions number of patients and the lesions uptake value scores of each patient information of 50 patients who did surgery

Items	Lesions number				Lesion uptake value scores of each patient		
Groups	1	2	3	4	1–3	4–6	7–9
Patients Number	1	4	6	39	27	12	10
Percentage (%)	2.00	8.00	12.00	78.00	55.10	24.49	20.41

**Fig. 5:**
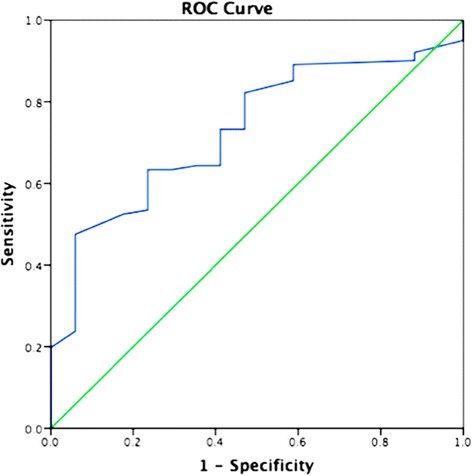
ROC analysis of diameter of lesion in SHPT patients

Following ROC analysis, the optimal cutoff values of the maximum tumor diameter for predicting positive ^99m^Tc-MIBI was 8.05 mm (AUC 0.731, 95% CI 0.618–0.843; sensitivity = 82.20% and specificity = 52.90%) (Fig. 5). The cutoff values of 8.05 mm was used for lesion diameter in the 118 lesions as measured by SPECT/CT. Eighteen lesions (66.67%) of 27 lesions were negative in ^99m^Tc-MIBI SPECT/CT in patients with a lesion diameter lower than the cutoff value (*P* < 0.0001) (Table 3).

**Table 3:** ^99m^Tc-MIBI SPECT/CT imaging in SHPT patients grouped according to the lesion diameter optimal cutoff value calculated by ROC analysis

	Diameter		χ^2^	*P* value
Groups	>8.05	≤8.05	40.60	<0.0001
MIBI (+)	83	9		
MIBI (−)	8	18		

## Discussion

We studied rare cases in patients with secondary hyperparathyroidism that all had renal failure. All the patients were undergoing both ^99m^Tc-MIBI dual phases SPECT/CT imaging and ultrasonography before surgery. ^99m^Tc-MIBI parathyroid imaging is a valuable method based on the differential washout rate between the thyroid tissue and parathyroid hyperplasia [12]. Parathyroid hyperplasia has very high metabolic rate despite its small size, and it shows intense ^99m^Tc-MIBI uptake. More recently, SPECT/CT systems combining state-of-the-art multi-detector CT and state-of-the-art gamma cameras are being produced, and guidelines for image acquisition, interpretation, and reporting for patients with SHPT have been published [13, 18]. SPECT/CT imaging also influences the surgical approach, enabling to choose different surgical approaches for lesions in different areas [19]. In our study, imaging detected an ectopic parathyroid in one patient. The major significance of ^99m^Tc-MIBI imaging pre-surgery was the detection of ectopic parathyroid, which guided the surgery. In our study, the sensitivities of ^99m^Tc-MIBI imaging on a per-patient basis and on a per-lesion basis were 98.00% and 59.34%, respectively. For USG the results were 78.00% and 46.24%, respectively.

In our study the sensitivity of ^99m^Tc-MIBI imaging on a per-lesion basis was 59.34% that exactly matches to a meta-analysis publication on planar ^99m^Tc-MIBI imaging the pooled sensitivity was 58% in SHPT [20]. There were many researches results showed that the sensitivity of SPECT/CT should be higher than planar ^99m^Tc-MIBI imaging [21]. There was also study showed that single-phase SPECT/CT (early or delayed) had not significant in superior to dual-phase planar imaging for sensitivity. Dual-phase SPECT/CT had statistically significant in superior to single-phase SPECT/CT and superior to dual-phase planar imaging and dual-phase SPECT [22, 23]. Therefore, ^99m^Tc-MIBI imaging show a limitation in the diagnosis of lesions in patients with SHPT. Usually we used the ^99m^Tc-MIBI uptake value as the guide information to diagnose whether the parathyroid lesions were; and CT information only was used to confirm the diagnosis. However, it was very difficult to differentiate the lymph node and the tumors only by CT for the lesions without ^99m^Tc-MIBI uptake in SPECT imaging. ^99m^Tc-MIBI has a lower sensitivity in multiple gland disease [24]. But the mechanism is not clear at present. The pre-surgical localization determines the success rate of surgery and the risk of recurrence. Compared to patients with PHPT who mostly have only one lesion [8], most of the patients with SHPT have multiple lesions [9]. The surgical results in our research also proved that 78% of the patients had four lesions. If the surgeon determines the surgical resection according to the ^99m^Tc-MIBI imaging results, many lesions would be missed. Intraoperative PTH assay is routinely used in clinical practice, and Ohe’s study showed that the decrease in the level of intraoperative PTH could predict the rate of successful parathyroidectomy [25].

In the per-lesion-based analysis, the specificity of ^99m^Tc-MIBI imaging was 75.00%. The most common cause false-positive results were the presence of thyroid nodules. Because MIBI can be taken up by both hyper-functioning parathyroid and thyroid tissue, the differential diagnosis is based on its elimination from the hyper-functioning thyroid and parathyroid glands. Normal parathyroid tissue without uptake in either of the two phases. The possibility of clearances in similar times for both tissues, which could lead to false negatives, has also been described [11, 26]. Specificity of parathyroid imaging is highly dependent on the prevalence of thyroid nodular disorders in the evaluated patient population [23]. The factors may affect the diagnostic accuracy rate were the followings: the location of parathyroid glands, the size of the lesion, and the functional activity of the lesions, and the prevalence of mitochondria-rich oxyphil cells [27, 28]. Lesion size is important as it relates to the system resolution and to the amount of tracer taken up by the parathyroid tissue [29]. Our results showed that lesion diameter was a statistically significant predictive factor in predicting positive ^99m^Tc-MIBI SPECT/CT. The larger the lesion diameter, the higher was the MIBI uptake value.

Serum PTH is a major regulator of mineral metabolism that plays a critical role in the maintenance of calcium and phosphate levels [30]. The major physiologic function of PTH is the circulation of ionized calcium. PTH has effects on the gut, kidney, and bone, serving to maintain serum calcium level within a tight range [31]. The pathogenesis of patients with PHPT is independent secretion of PTH by the parathyroid tissue. Unlike patients with PHPT, patients with SHPT have a compensatory PTH secretion for hypocalcaemia. Therefore, in patients with SHPT, the serum calcium level is uncertain; it may increase or remain normal. Pearson correlation analysis between PTH level and various factors showed that the serum AKP level and PTH level exhibit a significant linear association. PTH increased the bone metabolism conversion to elevate the serum AKP level. High level of AKP is the marker of increased bone conversion [32].

## Conclusion

Our study demonstrated that the dual phase ^99m^Tc-MIBI SPECT/CT imaging had a higher sensitivity in the diagnosis of patients with SHPT than ultrasonography on a per-patient-based analysis (98.00% vs. 78.00%). On a per-lesion-based analysis, the sensitivity and specificity of ^99m^Tc-MIBI were 59.34% and 75.00%, respectively. The results of ultrasonography were 46.24% and 80.00%. Dual phase ^99m^Tc-MIBI scan for positioning the lesion is an effective pre-surgical tool for patients with SHPT. The lesion diameter maybe a limitation for ^99m^Tc-MIBI SPECT/CT imaging sensitivity, when the lesion diameter was above 8.05 mm, the higher positive result can be obtained. Serum AKP and PTH level exhibited a significant linear association. That means the higher PTH level, the higher AKP level.

## Abbreviations

^99m^Tc-MIBI: Technetium-99 m-hexakis [2-methoxy-2-methylpropylisonitrile]
PHPT: Primary hyperparathyroidism
PTH: Parathyroid hormone
ROC: Receiver-operating characteristic
SD: Standard deviation
SHPT: Secondary hyperparathyroidism
USG: ultrasonography
